# Detection of *Escherichia coli* O157:H7 in Ground Beef Using Long-Read Sequencing

**DOI:** 10.3390/foods13060828

**Published:** 2024-03-08

**Authors:** Katrina L. Counihan, Siddhartha Kanrar, Shannon Tilman, Joseph Capobianco, Cheryl M. Armstrong, Andrew Gehring

**Affiliations:** Eastern Regional Research Center, United States Department of Agriculture, Agricultural Research Service, Wyndmoor, PA 19038, USA; siddhartha.kanrar@usda.gov (S.K.); shannon.tilman@usda.gov (S.T.); joseph.capobianco@usda.gov (J.C.); cheryl.armstrong@usda.gov (C.M.A.); andrew.gehring@usda.gov (A.G.)

**Keywords:** foodborne pathogens, method development, whole-genome sequencing, virulence genes, food testing

## Abstract

Foodborne pathogens are a significant cause of illness, and infection with Shiga toxin-producing *Escherichia coli* (STEC) may lead to life-threatening complications. The current methods to identify STEC in meat involve culture-based, molecular, and proteomic assays and take at least four days to complete. This time could be reduced by using long-read whole-genome sequencing to identify foodborne pathogens. Therefore, the goal of this project was to evaluate the use of long-read sequencing to detect STEC in ground beef. The objectives of the project included establishing optimal sequencing parameters, determining the limit of detection of all STEC virulence genes of interest in pure cultures and spiked ground beef, and evaluating selective sequencing to enhance STEC detection in ground beef. Sequencing libraries were run on the Oxford Nanopore Technologies’ MinION sequencer. Optimal sequencing output was obtained using the default parameters in MinKNOW, except for setting the minimum read length to 1 kb. All genes of interest (*eae*, *stx1*, *stx2*, *fliC*, *wzx*, *wzy*, and *rrsC*) were detected in DNA extracted from STEC pure cultures within 1 h of sequencing, and 30× coverage was obtained within 2 h. All virulence genes were confidently detected in STEC DNA quantities as low as 12.5 ng. In STEC-inoculated ground beef, software-controlled selective sequencing improved virulence gene detection; however, several virulence genes were not detected due to high bovine DNA concentrations in the samples. The growth enrichment of inoculated meat samples in mTSB resulted in a 100-fold increase in virulence gene detection as compared to the unenriched samples. The results of this project suggest that further development of long-read sequencing protocols may result in a faster, less labor-intensive method to detect STEC in ground beef.

## 1. Introduction

Foodborne pathogens, such as *Escherichia coli*, *Salmonella* spp., *Campylobacter* spp., and *Listeria monocytogenes*, remain a major cause of disease globally [1]. The World Health Organization estimates that each year, one in ten people worldwide will be sickened by a foodborne pathogen, and 420,000 people will die [2]. In the United States (U.S.), an estimated 48 million people become ill each year [3]. Additionally, pathogen contamination of food is a significant economic burden estimated to cost the world economy USD 110 billion [2] and the U.S. economy USD 17 billion annually [4]. During 2021, over 15 million pounds of meat were recalled in the U.S., and Shiga toxin-producing *Escherichia coli* (STEC) was the cause of two of those recalls, totaling 300,096 pounds [5]. Infections by STEC have been increasing since 2018 and have an incidence rate of 5.7 per 100,000 people [6]. An STEC infection generally causes diarrhea and vomiting but may result in severe diseases such as hemorrhagic colitis or hemolytic uremic syndrome [7].

The isolation and identification of STEC as an adulterant in meat by the U.S. Department of Agriculture Food Safety and Inspection Service (USDA FSIS) is achieved through a combination of culturing, molecular methods, O typing, and matrix-assisted laser desorption/ionization time-of-flight (MALDI-TOF) mass spectrometry [8]. Food samples are considered adulterated if they test positive for the *stx* and *eae* genes and one of the seven targeted O antigens commonly associated with STECs isolated from symptomatic patients [8]. The *stx1* and *stx2* genes encode Shiga toxins 1 and 2, which cause cytotoxicity in the host, and expression of *eae* produces intimin, which mediates enterocyte colonization [7]. The serotype most frequently associated with foodborne STEC outbreaks is O157:H7 [6]. The serotype of *E. coli* is determined by the H antigen, which is present on the flagella, and the O antigen, which is present on the outer membrane [9]. The current culture-based identification process takes at least four days to complete [8]. Whole-genome sequencing could reduce the amount of time and labor needed for foodborne pathogen identification.

Advances in whole-genome sequencing technology have led to third-generation, or long-read, sequencing that could significantly reduce the amount of time needed to identify foodborne pathogens compared to the current culture-based methods. Oxford Nanopore Technologies’ MinION device sequences RNA or DNA by detecting changes in electrical current as the strands of nucleic acid pass through nanopores on a flow cell [10]. The long reads generated facilitate genome assembly [11], while their real-time analysis allows pathogen detection to be accomplished in hours instead of days [12]. The small, portable sequencers allow whole-genome analysis to be conducted outside of traditional laboratories, and the cost is generally lower than second-generation sequencing. Additionally, significant progress has been made to reduce errors in nanopore sequencing, and raw read accuracy has improved to >99.9% [13]. A previous in silico study by our research group also suggested that long-read sequencing would be practical for testing food for *E. coli* and *L. monocytogenes* contamination after growth enrichment [14].

The goal of this project was to evaluate the potential of long-read whole-genome sequencing for STEC detection. The objectives included establishing optimal sequencing parameters, determining the limit of detection of all STEC virulence genes of interest in pure culture and STEC-inoculated ground beef, and assessing the ability of software-controlled enrichment and depletion of specific genomic material to enhance the detection of STEC in inoculated meat.

## 2. Materials and Methods

### 2.1. Bacterial Culturing

*Escherichia coli* O157:H7 (ATCC 43895; American Type Culture Collection, Manassas, VA, USA) was grown in tryptic soy broth (Oxoid Limited, Hampshire, UK) modified with novobiocin (mTSB; RPI Corporation, Mount Prospect, IL, USA) overnight at 42 ± 1 °C according to USDA’s Food Safety and Inspection Service procedures [8]. The optical density at a wavelength of 600 nm (OD 600) was measured with a DeNovix DS-11 FX+ spectrophotometer (DeNovix Inc., Wilmington, DE, USA) to determine bacterial concentration.

### 2.2. STEC Inoculated Ground Beef

Ground beef spiked with *E. coli* O157:H7 was processed according to USDA FSIS methods [8]. The following treatments were included: media only; uninoculated meat; 1 × 10^7^ cfu mL^−1^ *E. coli*; and ground beef inoculated with 1 × 10^5^ cfu g^−1^, 1 × 10^6^ cfu g^−1^, or 1 × 10^7^ cfu g^−1^ of *E. coli*. Treatments were prepared by placing 1 ± 0.01 g of ground beef on one side of a sterile 7 oz Whirl-Pak^®^ (Austin, TX, USA) strainer bag (except for the media and *E. coli* only controls) and then diluting 1:4 with mTSB. Each bag was stomached for 120 s with a Bag Mixer (Spiral Biotech Inc., Norwood, MA, USA). One experiment without enrichment was conducted with triplicate samples, and one experiment was conducted with enrichment with triplicate samples. The bags in the enrichment experiment were incubated statically for 24 h at 42 ± 1 °C. The samples were filtered through a 40 µm cell strainer (Greiner Bio-One North America Inc., Monroe, NC, USA) and then centrifuged at 130× *g* for 10 min. The supernatant was retained. An aliquot was plated on rainbow agar (Biolog Inc., Hayward, CA, USA) modified with potassium tellurite (Thermo Fisher Scientific, Waltham, MA, USA), novobiocin (RPI), and cefixime (RPI) (mRBA) to determine the concentration of *E. coli*. The remaining supernatant was centrifuged at 3400× *g* for 20 min. The supernatant was discarded, and the pellet was washed in 1 mL of phosphate-buffered saline (PBS; Boston Bioproducts Inc., Ashland, MA, USA) and then centrifuged at 12,000× *g* for 1 min. The supernatant was discarded, and the pellet was retained for DNA extraction.

### 2.3. DNA Extractions

The DNA from pure cultures of *E. coli* was extracted with a Monarch High-Molecular-Weight DNA Extraction Kit (New England BioLabs Inc., Ipswich, MA, USA) according to the manufacturer’s instructions. The pellets from the ground beef experiments were extracted using a Qiagen DNeasy PowerFood Microbial Kit (Qiagen, Germantown, MD, USA) according to the manufacturer’s instructions. DNA concentration and quality measurements were taken with a Denovix DS-11 FX+ spectrophotometer.

### 2.4. MinION Sequencing

Libraries of *E. coli* O157:H7 DNA were prepared using a Field Sequencing Kit (SQK-LRK001, Oxford Nanopore Technologies [ONT], Oxford, UK) according to the manufacturer’s instructions. A flow cell check was performed prior to sequencing to ensure that enough pores were available for sequencing. The MinION Mk1B or Mk1C (ONT) were used with R9 flow cells (ONT). The optimal sequencing run time for the DNA extracted from a pure culture of *E. coli* O157:H7 was determined by testing the following time points in triplicate: 1 h, 2 h, 4 h, and 6 h. The limit of detection (LOD) for identifying target virulence genes in *E. coli* O157:H7 DNA was determined by testing the following DNA concentrations in triplicate: 400 ng, 200 ng, 100 ng, 50 ng, 25 ng, 12.5 ng, 6.25 ng, 3.125 ng, 1.56 ng, 0.78 ng, and 0.39 ng. The 400 ng concentration was sequenced with a 1 h run duration, and the 200 ng concentration was sequenced with a 2.5 h duration. The duration of the sequencing run for the remaining concentrations was determined using nonlinear regression to approximate the amount of time needed to obtain 400 k reads, which was the average number of reads generated during a 1 h run time with optimal (400 ng) DNA input. The DNA from spiked ground beef experiments was sequenced for 24 h. In some of the meat sequencing runs, the software-controlled depletion of the *Bos taurus* (domestic bovine) genome or the enrichment of the *E. coli* O157:H7 genome was employed. The reference *B. taurus* genome (NCBI Accession #NC037338.1) was uploaded into the MinKNOW software (ONT, version 22.03.6), and software-controlled depletion was enabled. When software-controlled enrichment was enabled, the *E. coli* O157:H7 reference genome (NCBI Accession #NC002695.5) was uploaded into the MinKNOW software.

### 2.5. Quantitative Real-Time Polymerase Chain Reaction

Quantitative real-time polymerase chain reaction (qPCR) was used to confirm the presence of the *fliC*, *stx*, *eae*, and *rrsC* genes from *E.coli* O157:H7 following established USDA FSIS protocols [15]. A StepOne Real-Time PCR System (Applied Biosystems, Waltham, MA, USA) or QuantStudio 5 Real-Time PCR System (Applied Biosystems) was used for qPCR.

### 2.6. Data Analysis

Sequencing data were basecalled in real-time or post-run with MinKNOW software using fast or high-accuracy basecalling and a minimum read length filter of 1 kb. The FastQ files were imported into Geneious Prime software (version 2023) and aligned to an *E. coli* O157:H7 reference genome (NCBI Accession #NC002695.5) using Minimap2 (version 2.24). The target genes, namely *fliC*, *eae*, *stx1a*, *stx1b*, *stx2a*, *stx2b*, *rrsC*, *wzx*, and *wzy*, were searched for in the alignment, and the number of times each gene was detected was recorded. The mean, standard deviation, or standard error of triplicate runs were determined for sequencing run parameters, target gene detection, and qPCR Ct values.

## 3. Results

### 3.1. Optimal Sequencing Parameters

The optimal sequencing output was obtained using the default settings in MinKNOW, with the exception of the minimum read length, which was set to 1 kb. For DNA extractions from pure cultures of *E. coli* O157:H7, a sequencing run time of 1 h was sufficient to detect the targeted virulence genes an average of 30.18 times (Table 1). However, due to variability between sequencing runs, as can be seen in the standard deviations in Table 1, a run time of 3 h was selected for the sequencing of pure bacterial cultures to ensure that enough data were generated.

### 3.2. Limit of Detection

**Figure 1:**
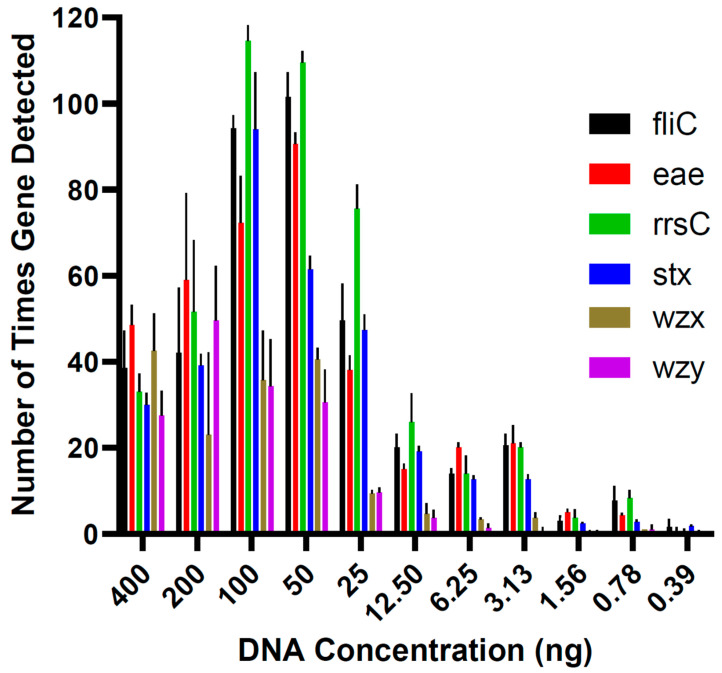
The mean ± standard error of the number of times each virulence gene of interest was detected in the *Escherichia coli* O157:H7 DNA concentrations tested in the limit of detection assays.

**Figure 2:**
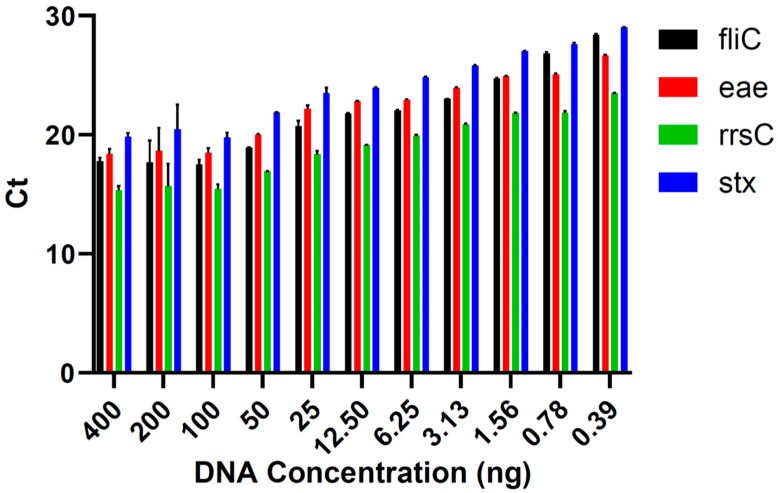
The mean ± standard error of the Ct values for each *Escherichia coli* O157:H7 virulence gene of interest detected using qPCR in the DNA concentrations used in the limit of detection assays.

In the runs conducted to determine the limit of detection in pure culture, the lowest DNA concentration at which all virulence genes of *E. coli* O157:H7 DNA were detected in each triplicate was 12.5 ng (Figure 1). Five lower concentrations (6.25, 3.13, 1.56, 0.78, and 0.39 ng) were tested, but all virulence genes could not be identified in all triplicates. Therefore, 12.5 ng was the lowest concentration of DNA that could be used to determine the *E. coli* serotype. The genes *fliC*, *eae*, *rrsC*, and *stx* were detected in the qPCR assay in all the DNA concentrations tested in the limit of detection analysis (Figure 2).

### 3.3. Spiked Ground Beef

**Figure 3:**
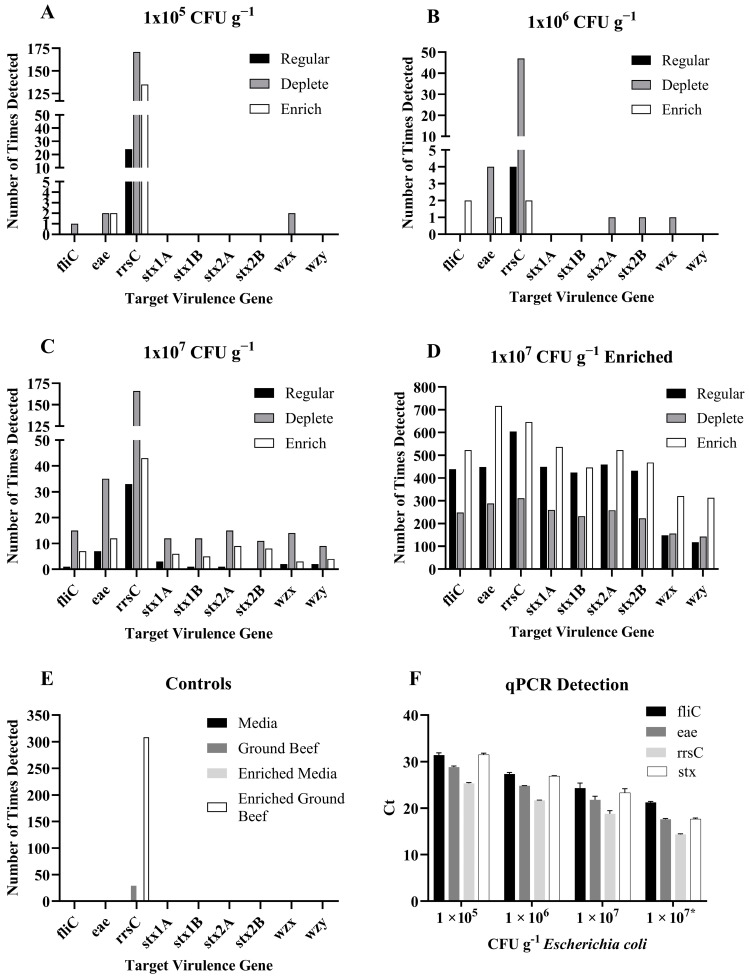
The number of times each virulence gene of interest was detected using regular, software-controlled depletion, or software-controlled enrichment long-read sequencing of ground beef inoculated with (**A**) 1 × 10^5^ CFU g^−1^ *Escherichia coli* O157:H7, (**B**) 1 × 10^6^ CFU g^−1^ *E. coli* O157:H7, (**C**) 1 × 10^7^ CFU g^−1^ *E. coli* O157:H7, and (**D**) 1 × 10^7^ CFU g^−1^ *E. coli* O157:H7 growth enriched for 24 h. The number of times each gene of interest was detected in (**E**) the media only, ground beef only, and media or ground beef only controls growth enriched for 24 h; (**F**) shows the mean ± standard deviation of Ct values in the qPCR assay for the virulence genes; * indicates the growth enrichment.

Only the *rrsC* gene was detected in the normal sequencing runs of ground beef spiked with 10^5^ or 10^6^ cfu g^−1^ *E. coli* O157:H7, and using the software-controlled depletion of the bovine genome or the enrichment of the *E. coli* O157:H7 genome did not significantly increase gene detection (Figure 3A,B). All the target genes, except *stx2B*, were detected in the 10^7^ cfu g^−1^ *E. coli* O157:H7-inoculated ground beef, and software-controlled enrichment significantly increased the detection of virulence genes, namely by two-fold (Figure 3C). Aliquots of each sample of spiked ground beef were also plated on agar prior to DNA extraction to determine the concentration of *E. coli* O157:H7 that remained after the stomaching, filtration, and centrifugation steps. The results indicated that there was a 1 log decrease in the concentration of *E. coli* O157:H7 from the amount inoculated in the meat to the amount present in the DNA extraction.

The low levels of virulence gene detection in the directly sequenced samples prompted the testing of ground beef inoculated with 10^5^, 10^6^, or 10^7^ cfu g^−1^ *E. coli* O157:H7 that was then growth-enriched for 24 h prior to DNA extraction and sequencing. Aliquots of each sample were plated after growth enrichment and prior to DNA extraction, and all inoculated meat samples contained 10^9^ cfu mL^−1^ *E. coli*. Therefore, only one set of samples, those subjected to the 10^7^ cfu mL^−1^ treatment, was sequenced. The target genes were detected hundreds of times, and software-controlled enrichment significantly increased detection. However, software-controlled depletion resulted in a significant decrease in detection for most genes (Figure 3D).

Uninoculated media and meat were included as controls in both the direct sequencing and 24 h enrichment experiments. The target genes were not detected in the media controls. The virulence genes were not detected in the uninoculated meat, but the *rrsC* gene was identified (Figure 3E). The *fliC*, *eae*, *rrsC*, and *stx* genes were detected in all *E. coli* O157:H7-inoculated meat samples via qPCR (Figure 3F).

## 4. Discussion

The results of this study suggest that long-read whole-genome sequencing has the potential to shorten the time needed for *E. coli* O157:H7 identification. Optimal sequencing parameters were established to provide the highest quality and quantity of sequencing data from pure cultures of *E. coli* O157:H7 and ground beef inoculated with *E. coli* O157:H7. The default settings in the MinKNOW software were sufficient, except for the minimum read length setting. The minimum read length was set to 1 kb to remove any reads shorter than that length from the sequencing data output. Reads longer than 1 kb were more likely to span enough of the gene for positive identification, and all targeted virulence genes were longer than 1 kb, except for the *stx* genes. However, *stx1a* and *stx1b* are adjacent on the chromosome, as are *stx2a* and *stx2b*, and the combined lengths of the genes exceed 1 kb (Table S1). The generation of short reads is likely due to the use of the Field Sequencing Kit for library preparation, which uses a transposase to cleave template DNA and add adapters for sequencing [16]. The Field Sequencing Kit was selected because it is fast (10 min) and requires minimal extra equipment (a heat block), which makes it better suited for use in on-site testing at a meat processing plant. However, the transposase can generate smaller fragments, especially in DNA that is already sheared. Short reads of less than 1 kb represented 10–50% of the total reads in the timed runs and had to be filtered out in the post-sequencing analysis. Setting the minimum read length at 1 kb in the MinKNOW software removed the short reads and eliminated this post-analysis step. Other library preparation kits are available that do not use a transposase, which would likely reduce the proportion of smaller fragments, but it would be at the expense of time and portability.

Sequencing data can be basecalled using Guppy, the integrated basecaller in the MinKNOW software, with three different models: fast, high accuracy, or super accuracy. A comparison of the fast and high-accuracy models was conducted with the run-time and meat sequencing experiment data. The super-accuracy model required a large amount of computing power and needed to be run on a high-performance computing cluster. Therefore, it was not included in the comparison because it would be impractical for on-site testing. Basecalling was typically completed in real time with the fast model, while high accuracy took over 24 h to complete. In the run-time experiments, an average of 98.63% of reads aligned to the *E. coli* O157:H7 reference genome, and high-accuracy basecalling only improved accuracy by 0.65%. In the meat experiments, fewer reads aligned to the reference genome due to the presence of bovine DNA, but high accuracy basecalling only improved the average percent of reads aligned from 16.31% to 16.66%. Target gene identification was not significantly improved for the run-time or meat experiments either. While the use of higher-accuracy models would be important in sequencing experiments designed to find small genetic changes, such as single-nucleotide polymorphisms (SNPs) [17], that level of accuracy was not needed to identify the virulence genes of interest. Therefore, the fast model was used because data were basecalled in real time and could be immediately analyzed to provide results on foodborne pathogen presence more quickly. The recent release of an accelerated basecaller, Dorado, by ONT, doubles the basecalling speed [18]. This could make higher-accuracy models more practical for real-time analysis in future studies.

A 1 h sequencing run time was sufficient to detect the virulence genes of interest in DNA extractions from pure cultures of *E. coli* O157:H7. However, variability was high between the independent replicate samples in both the timed runs and the limit-of-detection sequencing runs (Table 1). Other studies have also noted issues with inter-run variability [19,20]. Efforts were made to reduce variability between runs by using the same DNA extraction and having the same technician perform the experiments. The primary variable between runs was the flow cell (R9.4.1). Flow cells have a total of 2048 nanopores, but the number of pores available for sequencing varied between flow cells and was lower if the flow cell was being reused. However, an analysis of the timed runs and the meat runs found no correlation between pore availability and the number of reads generated or data produced. The variability between runs, regardless of run time, prompted the selection of a 3 h run time for DNA extracted from pure cultures to ensure sufficient data generation despite potential variability in flow cell performance. High-quality genome assemblies require 30× coverage to ensure that the entire genome is sequenced and to distinguish errors from sequence variations [21]. The virulence genes of interest were detected an average of 30 times during 1 h runs and 58 times during 2 h runs, suggesting that a 3 h run time would generate sufficient coverage despite potential variability.

All *E. coli* have an indistinguishable core genome that has genes for housekeeping, metabolic, and transport functions [22]. The *rrsC* gene is a core gene, which is why it can only be used for identification to the species level. The accessory genome of *E. coli* contains genes that characterize the pathogenicity of specific pathotypes [22]. Therefore, to confirm the O157:H7 serotype, *eae*, *fliC*, *stx*, *wzx*, and *wzy* need to be identified in a sample. Serial dilutions of *E. coli* O157:H7 DNA were tested to determine the limit of detection for all virulence genes of interest. Some of the virulence genes could be detected in the lowest concentration tested, 0.39 ng, but the lowest concentration at which all virulence genes were detected in each triplicate was 12.5 ng. The recommended DNA input for the Field Sequencing Kit is 400 ng, and extracting DNA from a bacterial culture or meat sample provides sufficient DNA to input the recommended concentration into the library preparation. However, we anticipated the potential need to sequence DNA from only one or a few colonies isolated on selective agar, which would yield lower concentrations of DNA than extraction from a bacterial culture or meat sample. The ability to detect genes of interest from a colony would save time by eliminating the need to culture it to a higher concentration to meet the recommended DNA input concentration for the sequencing kits.

The identification of the *E. coli* O157:H7 virulence genes of interest was difficult in the ground beef matrix due to the high prevalence of bovine DNA. In ground beef inoculated with 10^5^ or 10^6^ cfu g^−1^ *E. coli* O157:H7, only the *rrsC* gene was detected. All virulence genes of interest, except *stx2B*, were identified in the 10^7^ cfu g^−1^ *E. coli* O157:H7-inoculated ground beef, but most genes were detected less than 10 times. Software-controlled enrichment and depletion improved the detection of the virulence genes, but it did not increase it enough to detect all of the genes needed to positively identify *E. coli* O157:H7. The infectious dose of *E. coli* O157:H7 is in the 10 s of cfu [23]; therefore, a testing method needs to be able to detect low concentrations of a pathogen to protect consumers. The inability to detect all virulence genes of interest, even at fairly high inoculum concentrations, prompted the testing of growth-enriched samples. The results showed that all virulence genes of interest were detected >100 times. Software-controlled enrichment further increased detection, but depletion generally decreased detection. This is likely because the concentration of *E. coli* DNA was higher than the bovine DNA after the growth enrichment, making depletion unnecessary. A previous in silico study conducted to determine the practicality of using long-read sequencing for foodborne pathogen detection indicated that growth enrichment would be necessary [14], and the results of the current study confirm that growth enrichment will be necessary to ensure the detection of very low concentrations of *E. coli* O157:H7 contamination in ground beef using sequencing.

Variability was noted in the number of times specific genes were detected in the samples, and this is likely due to differences in the gene copy number and the presence of nonpathogenic *E. coli*. The genes *eae* [24], *fliC* [25], *wzx*, and *wzy* [26] are single-copy genes. Generally, there is only one copy of each *stx* subtype gene, as well, but more than one copy can be present [27]. These genes were typically identified in lower numbers than *rrsC*, of which there are multiple copies on the chromosome [28]. Additionally, in the uninoculated ground beef controls, the *rrsC* gene of *E. coli*, which is specific to the species level, was detected. However, none of the target virulence genes were identified. These results indicated the presence of nonpathogenic *E. coli* in the ground beef samples, which would also increase the concentration of the *rrsC* gene but not the other virulence genes since they are only found in pathogenic strains. As discussed above, the accessory genes that define pathotypes are of primary interest in sequencing data to distinguish between nonpathogenic and pathogenic strains [22]. Selective growth enrichment prior to sequencing can amplify pathogenic bacteria to ensure that their detection is not masked by nonpathogenic strains.

## 5. Conclusions

Foodborne pathogen detection using sequencing has multiple advantages over culture-based methods. The procedure can be completed in three days, which is one day faster than the current method. Sample preparation and enrichment occur on day 1; DNA extraction, library preparation, and 24 h sequencing are started on day 2; and the results are analyzed on day 3. Sequencing is also not labor-intensive. After the enrichment, which is the same as the current FSIS method, DNA extraction, library preparation, and flow cell loading only take 2 h. Bioinformatic analysis is accomplished in less than 30 min. Sequencing the whole genome also provides the needed information for serotype determination and the identification of antibiotic resistance genes. Additionally, multiple pathogens could be targeted in a sequencing run. Long-read whole-genome sequencing shows promise as an efficient method for the detection of foodborne pathogens in ground beef.

## Figures and Tables

**Table 1:** Mean ± standard deviation of run parameters and target gene detection in timed runs.

Time	Read Length (kb)	N50 (kb)	% Aligned	*fliC*	*eae*	*rrsC*
1 h	6.5 ± 0.9	10.7 ± 0.5	98.1 ± 1.6	29.7 ± 17.5	38.3 ± 18.2	26.3 ± 12.2
2 h	6.6 ± 0.7	10.8 ± 0.7	98.6 ± 1.1	53.7 ± 27.1	73.3 ± 39.0	59.7 ± 28.7
4 h	6.5 ± 0.8	10.7 ± 0.8	98.8 ± 0.9	99.7 ± 57.4	136.3 ± 84.4	115.3 ± 69.0
6 h	6.5 ± 0.9	10.6 ± 0.8	99.0 ± 0.8	133.7 ± 72.8	179.3 ± 115.5	153.7 ± 86.2
**Time**	***stx1A***	***stx1B***	***stx2A***	***stx2B***	***wzx***	***wzy***
1 h	27.0 ± 7.9	24.0 ± 5.2	34.3 ± 15.0	34.3 ± 18.2	33.7 ± 17.5	24.0 ± 8.2
2 h	53.3 ± 23.1	49.7 ± 21.9	60.7 ± 22.0	58.7 ± 26.6	60.7 ± 32.0	56.3 ± 28.2
4 h	93.3 ± 52.9	87.3 ± 47.7	113.0 ± 61.6	109.3 ± 64.3	114.0 ± 70.5	109.0 ± 68.5
6 h	123.3 ± 66.7	115.3 ± 59.1	146.0 ± 80.9	142.0 ± 83.4	158.0 ± 93.6	150.0 ± 98.5

## Data Availability

The data presented in this study are openly available in USDA at https://doi.org/10.15482/USDA.ADC/25164449.v1 (accessed on 1 February 2024), reference number [29].

## Supplementary Materials

The following supporting information can be downloaded at: https://www.mdpi.com/article/10.3390/foods13060828/s1, Table S1: Genome location, length, and GC content of the *Escherichia coli* O157:H7 genes of interest.

## References

[1] (2024). Food Safety. https://www.who.int/news-room/fact-sheets/detail/food-safety.

[2] (2024). Estimating the Burden of Foodborne Diseases. https://www.who.int/activities/estimating-the-burden-of-foodborne-diseases.

[3] (2023). Foodborne Germs and Illnesses. https://www.cdc.gov/foodsafety/foodborne-germs.html.

[4] Hoffmann S., Ahn J. Economic Cost of Major Foodborne Illnesses Increased $2 Billion from 2013 to 2018. https://www.ers.usda.gov/amber-waves/2021/april/economic-cost-of-major-foodborne-illnesses-increased-2-billion-from-2013-to-2018/.

[5] (2022). Summary of Recall Cases in Calendar Year 2021. Food Safety. https://www.fsis.usda.gov/food-safety/recalls-public-health-alerts/annual-recall-summaries/summary-recall-cases-calendar-8.

[6] Delahoy M., Shah H., Weller D., Ray L., Smith K., McGuire S., Trevejo R., Walter E., Wymore K., Rissman T. (2023). Preliminary incidence and trends of infections caused by pathogens transmitted commonly through food—Foodborne diseases active surveillance network, 10 U.S. sites, 2022. Morb. Mortal. Wkly. Rep..

[7] Pakbin B., Bruck W., Rossen J. (2021). Virulence factors of enteric pathogenic *Escherichia coli*: A review. Int. J. Mol. Sci..

[8] (2021). Method Number 5C.02. Microbiology Laboratory Guidebook. https://www.fsis.usda.gov/news-events/publications/microbiology-laboratory-guidebook.

[9] Fratamico P., DebRoy C., Liu Y., Needleman D., Baranzoni G., Feng P. (2016). Advances in molecular serotyping and subtyping of *Escherichia coli*. Front. Microbiol..

[10] Nanopores (2022). How Nanopore Sequencing Works. https://nanoporetech.com/how-it-works.

[11] Loman N., Pallen M. (2015). Twenty years of bacterial genome sequencing. Nat. Rev. Microbiol..

[12] Jain M., Olsen H., Paten B., Akeson M. (2016). The Oxford Nanopore MinION: Delivery of nanopore sequencing to the genomics community. Genome Biol..

[13] (2024). Nanopore Sequencing Accuracy. https://nanoporetech.com/platform/accuracy/.

[14] Counihan K., Kanrar S., Tilman S., Gehring A. (2024). Evaluation of long-read sequencing simulators to assess real-world applications for food safety. Foods.

[15] (2019). PCR Platform Instructions for the Real-Time PCR Detection of Shiga Toxin Gene and H7 Gene in *E. coli* O157:H7. https://www.fsis.usda.gov/sites/default/files/media_file/2021-03/mlg-5-appendix-5.pdf.

[16] (2024). Field Sequencing Kit. https://store.nanoporetech.com/us/field-sequencing-kit.html.

[17] Hyeon J., Li S., Mann D., Zhang S., Li Z., Chen Y., Deng X. (2018). Quasimetagenomics-based and real-time-sequencing-aided detection and subtyping of Salmonella enterica from food samples. Appl. Environ. Microbiol..

[18] (2023). Oxford Nanopore Releases Update to MinKNOW™ Software to Accelerate Basecalling Analysis, Further Accelerating High-Throughput Projects, at Scale. https://nanoporetech.com/about-us/news/oxford-nanopore-releases-update-minknowtm-software-accelerate-basecalling-analysis.

[19] Tyler A.D., Mataseje L., Urfano C.J., Schmidt L., Antonation K.S., Mulvey M.R., Corbett C.R. (2018). Evaluation of Oxford Nanopore’s MinION Sequencing Device for Microbial Whole Genome Sequencing Applications. Sci. Rep..

[20] Oikonomopoulos S., Wang Y.C., Djambazian H., Badescu D., Ragoussis J. (2016). Benchmarking of the Oxford Nanopore MinION sequencing for quantitative and qualitative assessment of cDNA populations. Sci. Rep..

[21] Sims D., Sudbery I., Ilott N., Heger A., Ponting C. (2014). Sequencing depth and coverage: Key considerations in genomic analyses. Nat. Rev. Genet..

[22] Geurtsen J., de Been M., Weerdenburg E., Zomer A., McNally A., Poolman J. (2022). Genomics and pathotypes of the many faces of *Escherichia coli*. FEMS Microbiol. Rev..

[23] Teunis P., Ogden I., Strachan N. (2008). Hierarchical dose response of *E. coli* O157:H7 from human outbreaks incorporating heterogeneity in exposure. Epidemiol. Infect..

[24] McCrea J., Liu C., Ng L., Wang G. (2007). Detection of the *Escherichia coli* pathogenic gene eae with three real-time polymerase chain reaction methods. Can. J. Microbiol..

[25] Cabal A., Geue L., Gomez-Barrero S., Barth S., Barcena C., Hamm K., Porrero M.C., Valverde A., Canton R., Menge C. (2015). Detection of virulence-associated genes characteristic of intestinal *Escherichia coli* pathotypes, including the enterohemorrhagic/enteroaggregative O104:H4, in bovines from Germany and Spain. Microbiol. Immunol..

[26] Hong Y., Reeves P. (2014). Diversity of O-antigen repeat unit structures can account for the substantial sequence variation of wzx translocases. J. Bacteriol..

[27] Ashton P., Perry N., Ellis R., Petrovska L., Wain J., Grant K., Jenkins C., Dallman T. (2015). Insight into Shiga toxin genes encoded by *Escherichia coli* O157 from whole genome sequencing. PeerJ.

[28] Botina S., Sukhodolets V. (2006). Speciation in bacteria: Comparison of the 16S rRNA gene for closely related Enterococcus species. Russ. J. Genet..

[29] Counihan K.L., Kanrar S., Tilman S., Capobianco J., Armstrong C.M., Gehring A. (2024). Long-Read Sequencing Data from Pure Cultures of *Escherichia coli* O157:H7 and Ground Beef Inoculated with *E. coli* O157:H7. Ag Data Commons.

